# Numerical and Experimental Study on Balanced Performance and Axial Stiffness of Fiber-Reinforced Rubber Pipe

**DOI:** 10.3390/polym16142088

**Published:** 2024-07-22

**Authors:** Jingyue You, Yinglong Zhao, Ben Zhang

**Affiliations:** 1 Institute of Noise and Vibration, Naval University of Engineering, Wuhan 430033, China; 21100203@nue.edu.cn (J.Y.); 19500202@nue.edu.cn (B.Z.); 2 National Key Laboratory on Ship Vibration and Noise, Naval University of Engineering, Wuhan 430033, China

**Keywords:** fiber-reinforced rubber pipe, balanced performance, axial stiffness, finite element method

## Abstract

Balanced fiber-reinforced rubber (FRR) pipes not only provide displacement compensation when transporting pressurized media but also prevent additional forces and displacements from being exerted on the connected pipeline system. Investigating the balanced performance of FRR pipes and the axial stiffness of balanced pipes is crucial for optimizing pipeline design and improving the reliability of pipeline systems. This paper develops a numerical model of FRR pipes that considers the nonlinearity of the rubber material and the interaction between the rubber matrix and fiber-reinforced layers. Using this model, the balanced performance of the pipe is calculated, and its axial stiffness under combined internal pressure and axial load is analyzed. Numerical results are compared with experimental data for validation. The results indicate that the pipe’s balance is achieved through the combined effects of the elongation and rotation of the reinforcing fibers and the deformation of the rubber matrix, highlighting the significant impact of the rubber matrix on the mechanical performance of the FRR pipe. Furthermore, the pipe’s balanced performance and axial stiffness are highly sensitive to the winding angle of reinforcing fibers. The proposed numerical model fills the gap in using numerical methods to evaluate the balanced performance of FRR pipes and provides valuable insights for their design and optimization.

## 1. Introduction

Fiber-reinforced polymer (FRP) pipes are typically fabricated using polymers as the matrix and fibers as the reinforcing material [1]. The performance characteristics of these pipes can be customized by adjusting the type of polymers as well as the type, volume fraction, and winding angle of the fibers [2]. Compared to traditional steel pipes, FRP pipes exhibit outstanding chemical and physical properties such as corrosion resistance and a high strength-to-weight ratio. Consequently, they have found extensive applications in the petrochemical industry, marine engineering, and civil engineering [3,4,5,6]. Researchers have extensively investigated the mechanical properties of FRP pipes under various environmental conditions, including temperature [7,8], medium [9,10], and humidity [11]. Studies have explored pipes’ behavior under internal pressure loads [3,4], axial loads [12], radial loads [13], and combined external forces [14,15]. The fiber-reinforced rubber (FRR) pipe is one of such pipes.The balanced performance of FRR pipes has also garnered attention. The balanced performance index measures the axial deformation capacity of the pipe under internal pressure [16]. Pipes with good balanced performance can not only provide displacement compensation when transmitting pressurized media but also avoid exerting additional forces and displacements on connected equipments or pipelines. This balanced performance index represents a higher standard in the custom design of FRR pipes to meet specific application requirements compared to traditional criteria such as stiffness and failure.

In studying the balanced performance of FRR pipes, Gao et al. [17] utilize thin shell theory to investigate the effects of fiber winding angles and pipe curvature radii on the balanced performance of the pipe, disregarding the influence of the rubber matrix. Based on the finding of Jaszak et al. [18], which identified that the maximum stress in FRR pipes occurs in the fiber-reinforced layer, Xu et al. [19] similarly ignored the impact of the rubber matrix in their theoretical study of the axial and lateral stiffness of self-balancing FRR pipes. However, Fang et al. [20] use the finite element method to reveal that, despite the higher Young’s modulus of the reinforcing fiber, the larger volumetric proportion of the polymer matrix makes its influence on the mechanical properties of FRP pipes significant and non-negligible. Consequently, the impact of the rubber matrix on the balanced performance of FRR pipes has not been adequately addressed to date.

The “netting-analysis” method, which assumes that the internal pressure is borne solely by the reinforcing fibers and neglects the contribution of the matrix material, can be used to assess the stress state of FRP pipes [21]. This theory suggests that the optimal fiber winding angle is 54.7°. However, Evans and Gibson [22] point out that the accuracy of the “netting-analysis” results is valid only when the stiffness of the matrix is significantly lower than that of the reinforcing fibers. Nonetheless, they did not provide the specific stiffness ratio between the matrix and the reinforcing fibers for different materials. Gu et al. [23], utilizing the three-dimensional anisotropic elasticity theory, derive the analytical solutions for the stress and strain distribution in steel-wire-wound reinforced rubber pipes.They attributed the discrepancies between their theoretical and experimental results to the simplification of the rubber matrix as a linear elastic material. In addition, researchers have employed various analytical methods to study the mechanical behavior of fiber-reinforced composites. For randomly oriented graphene nanoplatelet-reinforced 2D notched epoxy plates, Kabir et al. use a meshless technique combining a Gaussian quadrature technique and a Bezier based multi-step method to investigate the stress of the plates [24]. Wang et al. apply the generalized finite difference method combined with domain decomposition techniques and the meshless generalized finite difference method for the stress analysis of 3D elastic composites [25]. The simulation results demonstrate that this method is accurate and efficient in numerical simulations of multi-layered materials.

Gao et al. [3] employ numerical methods utilizing the embedded element technique to simulate the embedding of reinforcing fibers within the rubber matrix. This study examines the effects of different fiber and rubber materials on pipes’ deformation, stress distribution, and failure mechanisms under internal pressure, accounting for the nonlinear characteristics of both the fiber and rubber materials. The reliability of the numerical results is verified through experiments. Similarly, Wei et al. [26] investigate the impact of fiber winding angles and twisting directions on the torsional stiffness of FRP pipes using numerical methods, considering the nonlinearity of materials. The numerical results are also validated against experimental findings. Moreover, the literature [27,28,29,30] includes in-depth analyses of the mechanical properties of FRP pipes, incorporating material nonlinearity into their numerical methods and validating these findings through experimental results. In conclusion, these studies indicate that the numerical method can accurately simulate the mechanical behavior of FRP pipes made from various polymers and fibers under different loading conditions. Furthermore, numerical methods enhance the intuitive and detailed understanding of deformation, stress, and strain, thereby serving as a valuable tool for studying the balanced performance of FRR pipes.

The winding angle of fibers is a critical parameter influencing the mechanical properties of FRP pipes [2]. Consequently, the balanced performance of pipes can be achieved through specific winding angles of fibers [19]. According to the literature [31], the sensitivity of a pipe’s leakage and fracture strength are significantly affected by the fiber winding angle. Krishnan et al. [32,33] employed experimental methods to study the failure modes of glass/epoxy pipes with fiber-reinforced layers wound at $\pm 45^{{}^{\circ}}$, $\pm 55^{{}^{\circ}}$, and $\pm 63^{{}^{\circ}}$ under multiaxial cyclic loads. The results demonstrated that the optimal winding angle differs for pure hydrostatic loading, hoop to axial loading, and quad hoop to axial loading. Beyond the investigation of single winding angles, Xia et al. [34] explore the axial and torsional strength of multi-layered filament-wound pipes under internal pressure, concluding that the stacking sequence of fiber-reinforced layers plays a crucial role in determining the stress and deformation of the pipes. Further studies have combined the winding angle of fibers with other structural parameters to explore the impact on the mechanical properties of FRP pipes under multivariable influences [35,36]. However, the influence of fiber winding angle on the balanced performance of FRR pipes has not been sufficiently explored in the existing literature.

The purpose of this study is to investigate the balanced performance and axial stiffness of the FRR pipe under internal pressure. To achieve this, experiments are conducted to reveal the structural response of the pipe under maximum working pressure, and to investigate the combined influence of internal pressure and axial load. To characterize the nonlinear mechanical properties of the rubber material, the strain energy density model is fitted based on deformation test results. Subsequently, a numerical model considering the interaction between the rubber matrix and reinforcing fibers is developed. The reliability of this numerical model is verified through experimental validation. Additionally, this study reveals the influence of fiber winding angles on the balanced performance and axial stiffness of the FRR pipe.

## 2. Experiment

### 2.1. Experimental Samples

**Figure 1:**
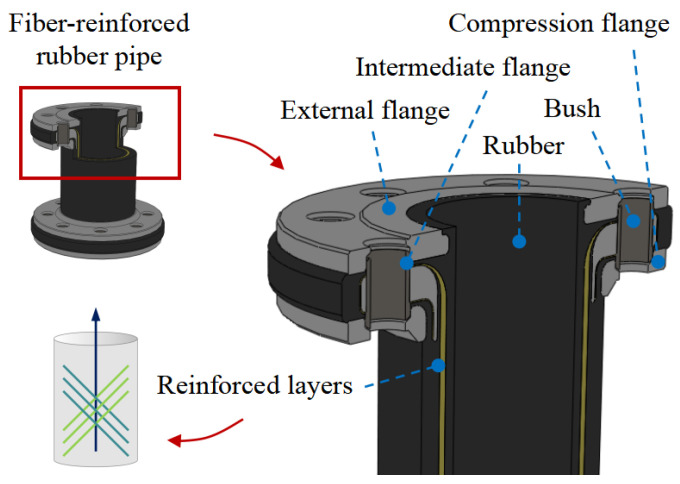
The structure of fiber-reinforced rubber (FRR) pipe.

**Figure 2:**
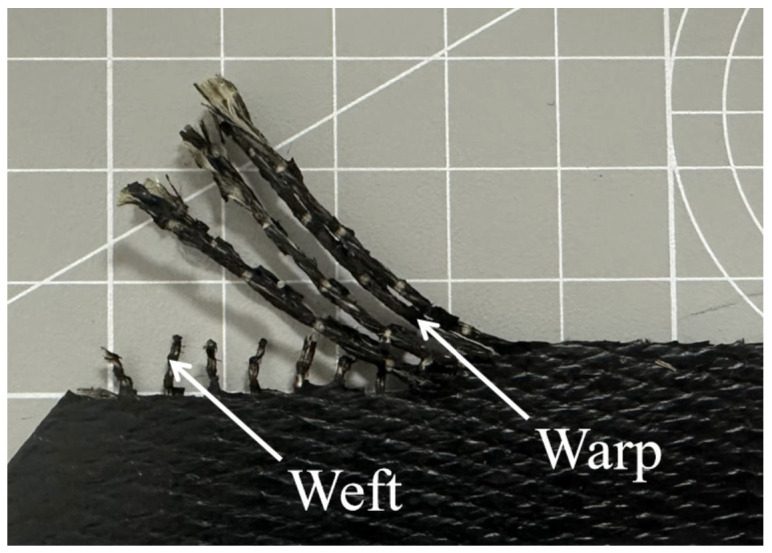
The rubber–fiber fabric.

The structure of the FRR pipe is illustrated in Figure 1. The primary components include flanges and fiber-reinforced polymers. The flanges are composed of a compression flange, an intermediate flange, and an external flange, which together ensure the joint’s resistance to pull-out forces. The fiber-reinforced polymers are produced by vulcanizing rubber with spirally wound reinforcing fibers, effectively bonding them together. This design prevents interfacial gaps and relative slippage between the rubber and reinforcing fibers. Specifically, as shown in Figure 2, the rubber–fiber fabric embedded in the rubber matrix is woven with warp and weft threads. The weft threads connect and protect the warp threads, ensuring their uniform arrangement within the fabric. During the manufacturing process, the fabric is wound around the intermediate flange and secured by the compression and external flanges, relying on installation force for locking.

**Figure 3:**
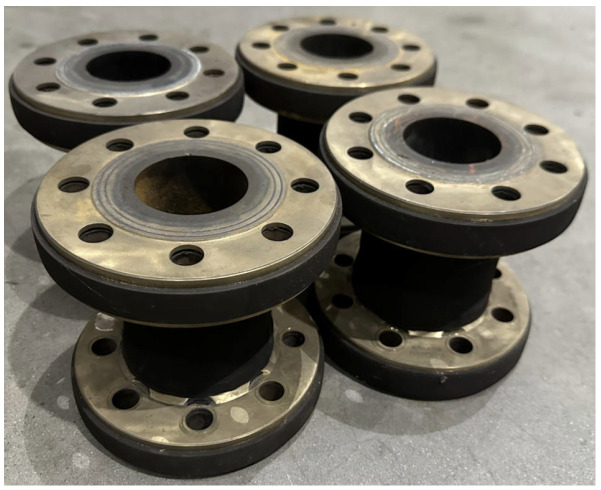
Test samples.

The test samples are shown in Figure 3, with a maximum working pressure of 1.6 MPa. To ensure the balanced performance of the pipes, a trial-and-error method is employed during the initial production stages to determine the balancing winding angle of ±36.9°. Further details on the manufacturing parameters can be found in Table 1.

### 2.2. Experimental Process

The measurement results for the length and outer diameter of the test samples are presented in Table 2. It is evident that discrepancies exist between the actual measurement results and the manufacturing parameters, with the maximum length deviation being 0.3 mm and the maximum outer diameter deviation reaching 5.73 mm. However, these discrepancies are relatively minor, indicating the robustness and stability of the production technique. Therefore, it is reasonable to assume that the structural parameters of the test samples are consistent with the manufacturing parameters, suggesting that the experimental results of the four specimens are comparable.

**Figure 4:**
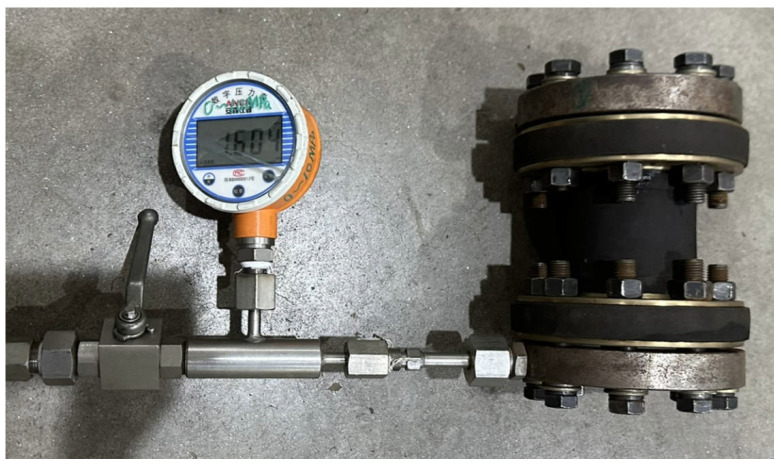
Balanced performance test of the FRR pipe.

The balanced performance and axial stiffness tests of the FRR pipe are conducted under ambient temperature and pressure conditions. The ultimate balanced performance of the pipe is investigated by conducting the balanced performance test at the maximum working pressure (1.6 MPa). To ensure the accuracy of the balanced performance test, the pipe is laid flat on the ground to minimize the impact of gravity on the flanges and end plates at both ends, as shown in Figure 4. After installing end plates, the initial length of the pipe is measured. Subsequently, nitrogen gas is slowly introduced into the pipe through a hole in one of the end plates until the internal pressure reaches the maximum working pressure. The length of the pipe is then measured again under pressurized conditions.

**Figure 5:**
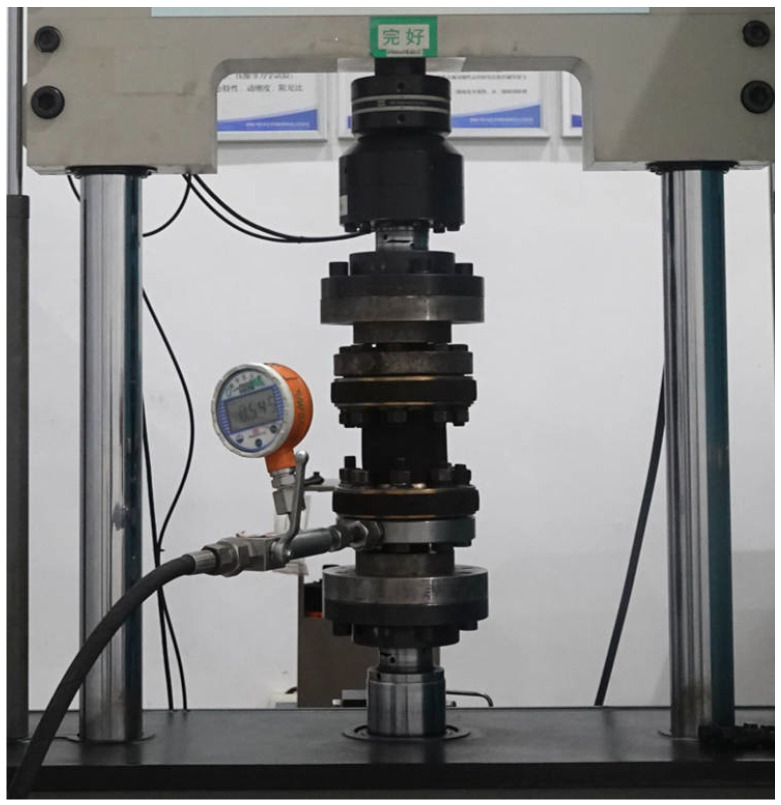
Axial stiffness test of the FRR pipe.

To better reflect the axial stiffness of the pipe under different working pressures in practical engineering applications, 0 MPa, 0.5 MPa, 1 MPa, and 1.5 MPa are chosen as the test pressures. The axial stiffness test is conducted using the MTS machine, as shown in Figure 5. The pipe is mounted on end plates, which are then installed onto the testing machine. Upon completion of the installation, the testing machine carries out load and displacement zeroing operations. During the test, nitrogen gas is slowly injected into the pipe to achieve the test pressures, after which the valve is closed. The testing machine applies a static load using displacement control, with an axial displacement peak set at ±2 mm and a loading rate of 0.1 mm/s.

### 2.3. Experimental Results

The axial deformation of the test samples under the maximum working pressure is listed in Table 3, where negative values indicate the shortening of the pipe under internal pressure. According to Reference [16], a pipe is considered balanced when the axial deformation under internal pressure is 0 mm, which is an idealized definition. In practical applications, Gao et al. [16] proposed that for the curved pipe they studied, an axial deformation of less than ±1 mm under maximum working pressure indicates that the pipe is balanced. According to this criterion, the maximum axial deformation of the pipe under maximum working pressure is 0.35 mm (as shown in Table 3), which is less than 1 mm, indicating that the test samples are balanced.

**Figure 6:**
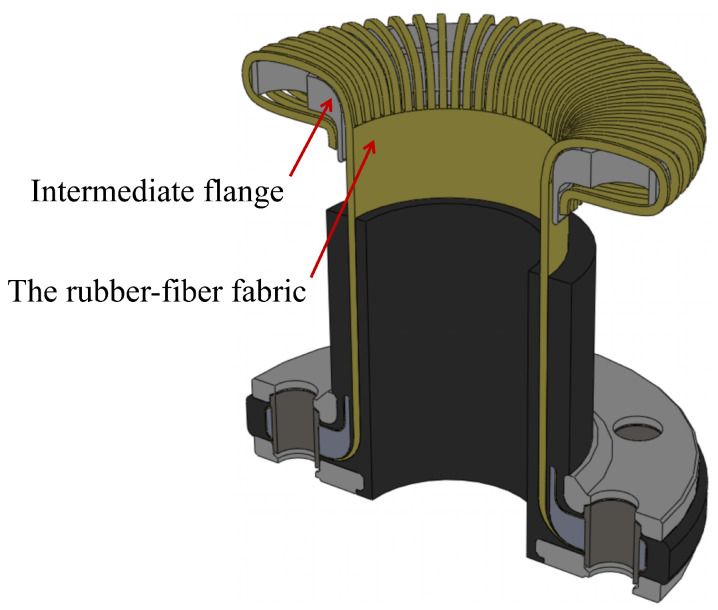
The rubber–fiber fabric is wound around the intermediate flange.

As shown in Table 3, under identical internal pressure values, different test samples exhibit either elongation or shortening. This variability is primarily attributed to the manual placement of the rubber–fiber fabric by operators during production. In accordance with the manufacturing process of the FRR pipe (as illustrated in Figure 6), operators first tear the entire piece of rubber–fiber fabric into narrow strips before pulling and adhering them to the intermediate flange when winding the fabric around it. This process may introduce additional tension and alter the fiber winding angle, resulting in inconsistent axial deformation of the pipe under maximum working pressure.

**Figure 7:**
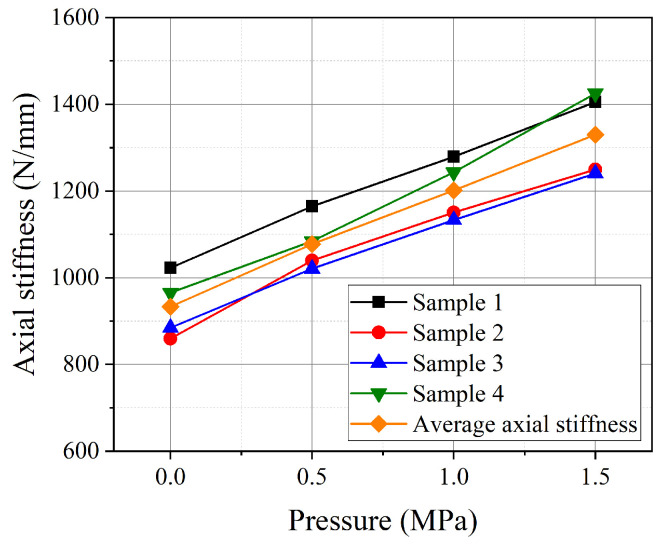
The axial stiffness of pipes under different internal pressures.

**Figure 8:**
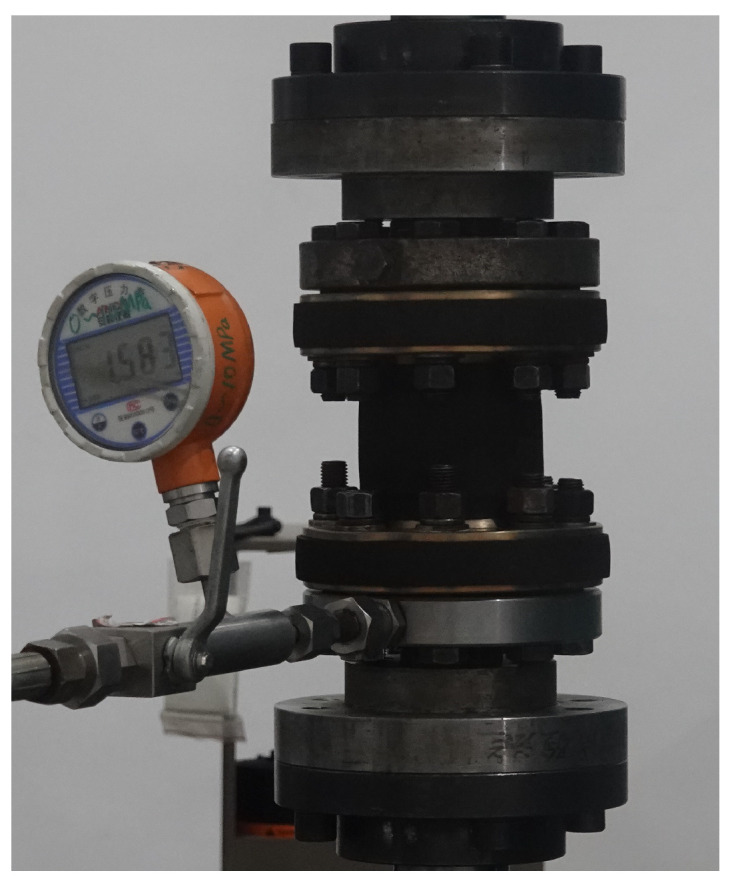
Expansion phenomenon of the FRR pipe under compression load and internal pressure.

Figure 7 illustrates the axial stiffness of the pipes under different internal pressures. It can be observed that the axial stiffness increases nonlinearly with rising internal pressure. The maximum error due to manufacturing factors appears in Sample 1. At an internal pressure of 0 MPa, the axial stiffness of Sample 1 is 1022.67 N/mm, whereas the average axial stiffness of the four pipes is 933.02 N/mm, resulting in a discrepancy of 8.77%. During the compression process, as the internal pressure increases, the expansion phenomenon of pipe is observed, as detailed in Figure 8.

## 3. Numerical Model

Using ABAQUS software version 2022, a three-dimensional nonlinear finite element model is established, with dimensions that are the same as the manufactured parameters. This approach ensures that the numerical results are comparable to the experimental results.

### 3.1. Geometric Model

**Figure 9:**
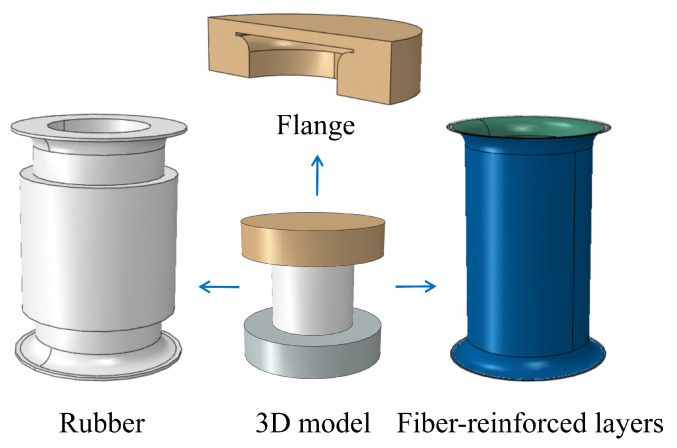
Simplified geometric model of the FRR pipe.

To enhance computational efficiency while maintaining structural integrity, the geometric model of the FRR pipe is simplified, as shown in Figure 9. The flanges and end plates, composed of aluminum bronze and carbon steel, exhibit significantly higher stiffness and much smaller deformation compared to other parts of the pipe. Therefore, these are modeled as rigid bodies. The rubber and fiber-reinforced layers, wound around the intermediate flange, are constrained by the installation force. This part of the structure can be ignored.

**Figure 10:**
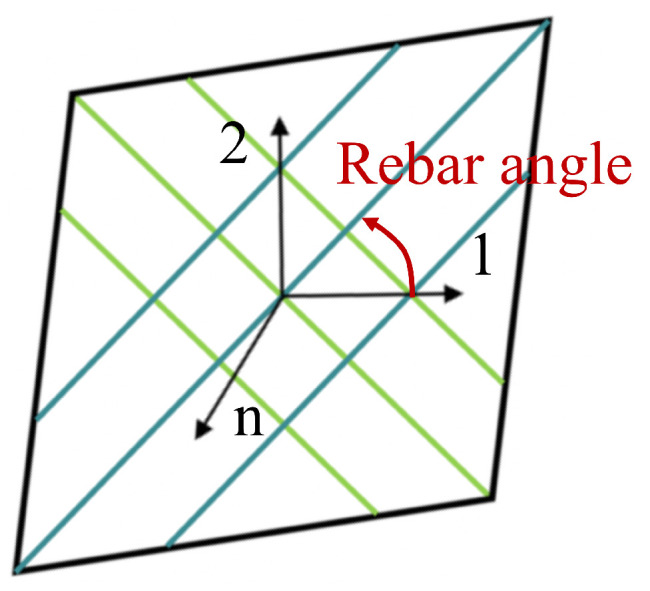
The local coordinate system of the rebar element.

The fiber-reinforced layers within the rubber matrix can be modeled using rebar elements. Each rebar element has a cross-sectional area of 0.636 mm^2^ and is spaced at intervals of 1.111 mm. The orientation angle of the rebar element is ±36.9°, which is defined as the angle between local direction 1 and the reinforced fibers, as illustrated in Figure 10.

### 3.2. Material Property

**Figure 11:**
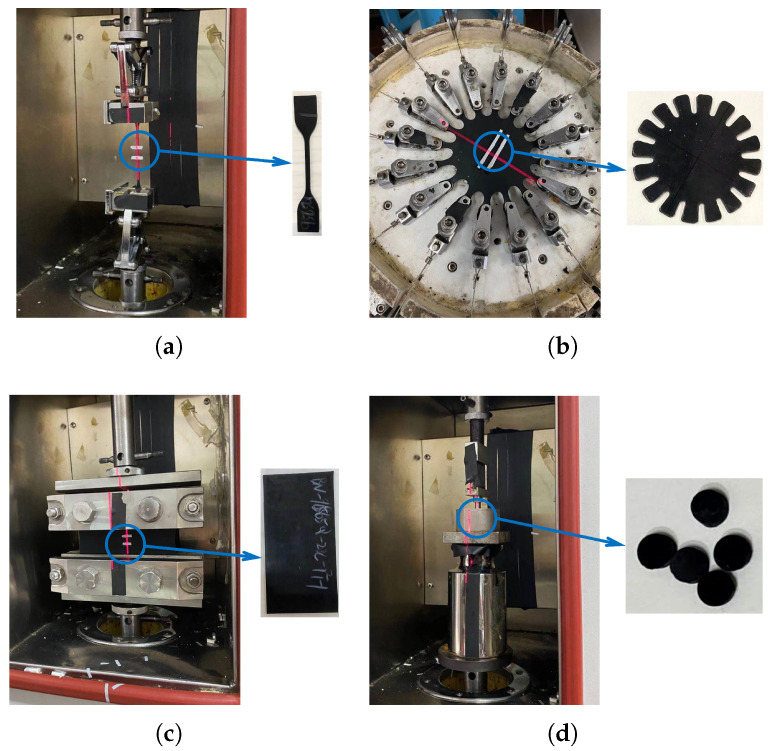
Test samples of the rubber material and testing process: (**a**) uniaxial tensile test; (**b**) equibiaxial tensile test; (**c**) planar tensile test; (**d**) volumetric compression test.

Rubber materials exhibit isotropic and incompressible characteristics, and their mechanical behavior can be described using strain energy density models [37]. Due to the Mullins effect, rubber materials undergo instantaneous and irreversible softening, which causes their stress–strain relationship to be influenced by the maximum historical load. Therefore, when fitting data for strain energy density models, the strain level used in the material test should correspond to the actual strain level experienced by the FRR pipe. During the preparation of the rubber samples, the vulcanization temperature and duration used are the same as those employed in the fabrication of FRR pipes. The samples and testing procedures are illustrated in Figure 11. For the uniaxial tensile test, a D-type dumbbell-shaped sample is used. In the case of the equibiaxial tensile test, a circular sample with a diameter of 60 mm and a thickness of 2 mm is utilized. For the planar tensile test, a rectangular sample with dimensions of 150 mm × 75 mm × 2 mm is selected. The volumetric compression test is conducted using a circular sample with a diameter of 8 mm.

**Figure 12:**
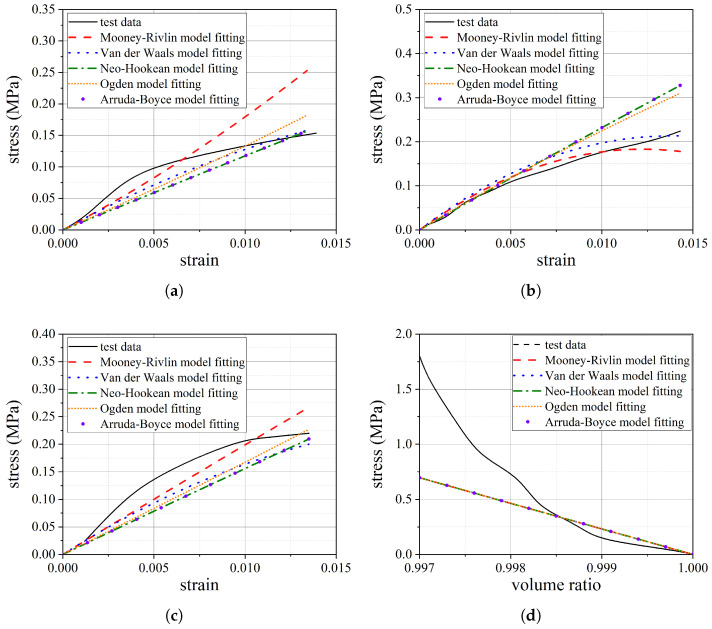
Test data fitting of rubber material: (**a**) uniaxial tensile fitting (**b**); equibiaxial tensile fitting (**c**); planar tensile fitting (**d**); volumetric compression fitting.

To evaluate the capability of various strain energy density models in characterizing the mechanical behavior of rubber materials, five common models are utilized for fitting. The fitting results are presented in Figure 12. During the fitting process, it is observed that the Mooney–Rivlin model and the Van der Waals model produce negative parameter values, which indicate potential material instability. In contrast, the parameter values for the other models are positive, ensuring material stability. Given the consistency of the fitting results among the other models, the neo-Hookean model is selected to describe the mechanical behavior of the rubber material. The parameters of the neo-Hookean model are obtained using ABAQUS software, where D1 is 8.62 × 10^−3^ and C10 is 1.98. In addition, the density of the reinforcing fiber is 1.44 × 10^−9^ t/mm^3^, Young’s modulus is 3.28 × 10^4^ MPa, and Poisson’s ratio is 0.36.

### 3.3. Mesh, Interaction, Load, and Boundary Condition

**Figure 13:**
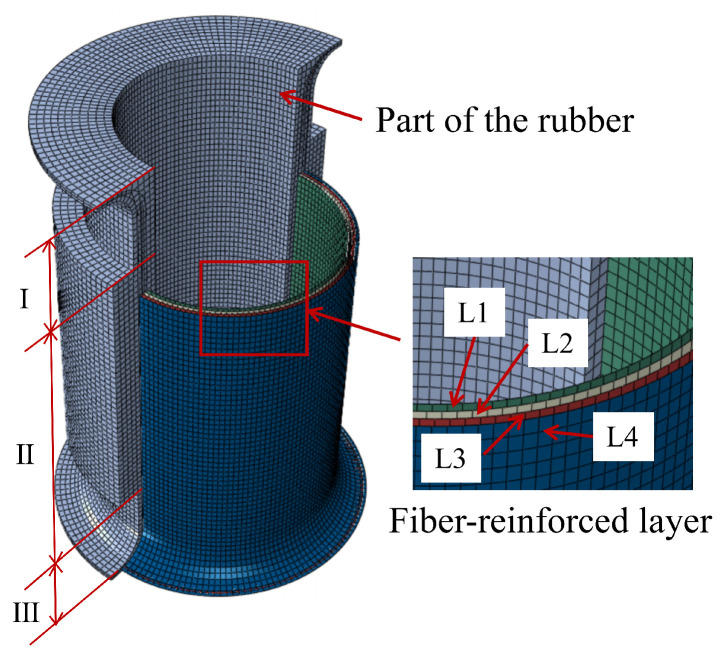
The finite element model of the pipe.

Following the mesh convergence analysis, the finite element model of the FRR pipe (as shown in Figure 13) is determined to consist of 131,616 elements. The rubber material is modeled using the C3D8R element, where ’R’ signifies reduced integration. Compared to fully integrated elements, the reduced integration technique shortens the computation time. The fiber-reinforced layers are modeled using four-node quadrilateral membrane elements (M3D4R).

**Figure 14:**
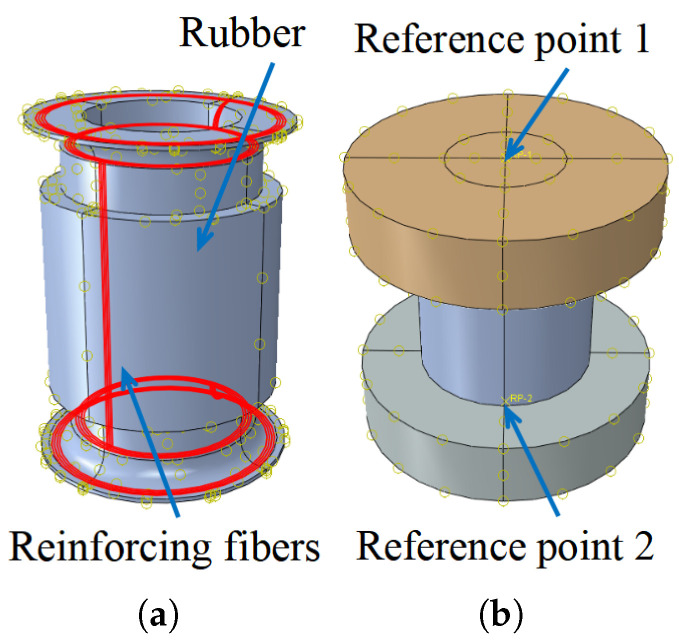
Interaction and reference points: (**a**) the reinforcing fibers embedded in the rubber matrix; (**b**) reference points.

Utilizing embedded element technology, as mentioned in reference [3], the simulation integrates the reinforcing fibers into the rubber matrix, as illustrated in Figure 14a. Reference points 1 and 2 are established on the surfaces of the upper and lower end plates, respectively. These reference points are rigidly constrained to the plates, effectively limiting the degrees of freedom of all plates’ nodes to these reference points, as illustrated in Figure 14b. During the balanced performance analysis, reference point 2 is fixed, and a uniformly distributed load is applied to the inner surfaces of the pipe. For the axial stiffness calculation, an additional ±2 mm axial displacement is applied to reference point 2, based on the boundary conditions established during the balanced performance analysis.

## 4. Results and Discussion

### 4.1. The Balanced Performance

**Figure 15:**
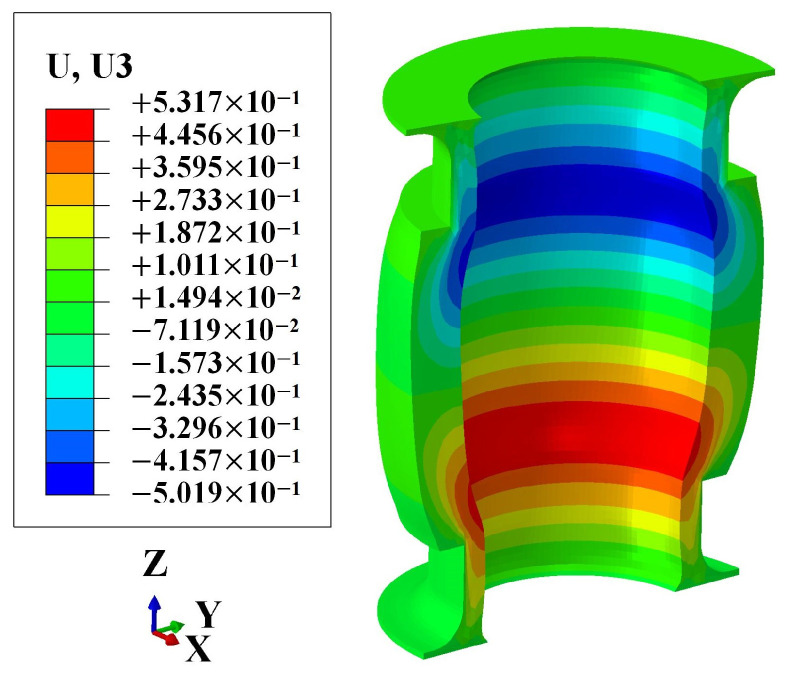
Axial deformation nephogram of the rubber.

The numerical results indicate that under maximum working pressure, the axial deformation of the FRR pipe is 0.03 mm. The numerical result is almost identical to the experimental results of Sample 1 and Sample 4 (as shown in Table 3). However, the absolute error for Sample 3 is 0.32 mm. This discrepancy accounts for only 0.19% of the total length of the pipe, which can be considered very small. Furthermore, the numerical and experimental results meet the requirement of axial deformation being less than 1 mm, indicating compliance with the balanced performance criteria. This demonstrates that the numerical result is within a reasonable error range, validating the reliability of the numerical method used to evaluate the balanced performance of the FRR pipe. Meanwhile, the numerical result shows that the axial deformation of the pipe under maximum working pressure is only 0.018% of the pipe length. This indicates that using balanced pipes in the pipeline system can prevent quality and safety risks caused by axial deformation exceeding the operational tolerance range due to internal pressure. To better illustrate the axial deformation of the pipe, the deformation coefficient in the nephogram is magnified tenfold, as shown in Figure 15. It is evident that under maximum working pressure, the pipe tends to expand radially, and the axial deformation at both sides of the pipe occurs in opposite directions.

**Figure 16:**
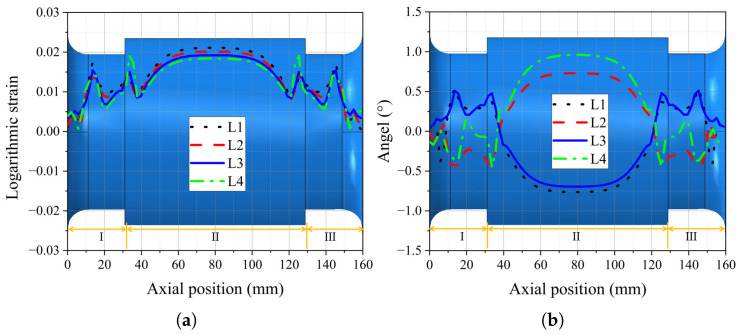
The logarithmic strain and rotation angles of each fiber-reinforced layer along the axial direction of the pipe: (**a**) logarithmic strain; (**b**) rotation angles.

**Figure 17:**
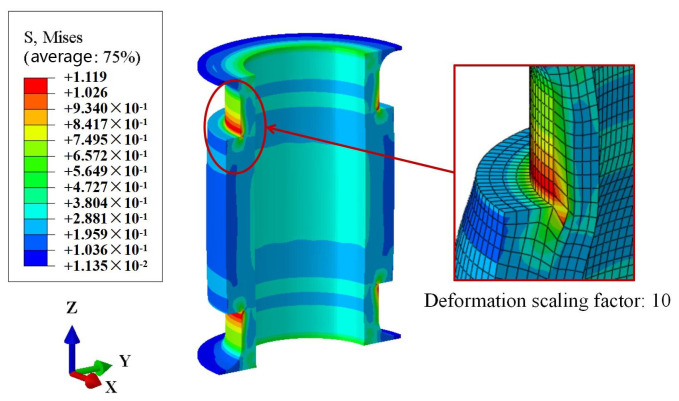
Mises stress nephogram of rubber.

By analyzing the logarithmic strain and rotation angles of each fiber-reinforced layer, a deeper understanding of the deformation and the balanced performance of the FRR pipe under maximum working pressure can be achieved. As shown in Figure 16a, all four fiber-reinforced layers exhibit elongation under internal pressure. The logarithmic strains in the L1, L2, and L3 layers decrease with an increasing radius in different regions of the pipe, which can be attributed to the “buffering” effect of the rubber dissipating the forces generated by the internal pressure. The L4 layer shows a different tendency from the other layers at the intersection of regions II and III. Because L4 is the outermost layer, its strain is affected by the rubber and the end plate. Where the rubber is attached to the end plates, caused by the constraint of end plates and the change in the rubber cross-section, obvious shear deformation is generated compared with other regions, as shown in Figure 17.

The rotation angles of the fiber-reinforced layers under maximum working pressure are depicted in Figure 16b. The initial orientation angles of the L1 and L3 layers are 36.9°. According to the local coordinate system shown in Figure 10, these layers primarily rotate towards coordinate axis 2 in regions I and III of the pipe, while in region II, they rotate towards coordinate axis 1. This behavior is reversed for the L2 and L4 layers because of their opposite initial orientation angles. This suggests that, under internal pressure, the fiber-reinforced layers experience radial tension in region II. In regions I and III, the fiber-reinforced layers are primarily subjected to axial tension, which is attributed to the constraining effect of the flanges on radial deformation and the end effect of the internal pressure on the end plates of the pipe. The rubber matrix embedding the L4 layer undergoes shear deformation, resulting in its rotation angles in regions I and III differing from those of the other layers. Additionally, as the outermost layer, its rotation angle in region II is significantly greater than that of the other fiber-reinforced layers. In summary, the elongation and rotation of the fiber-reinforced layers, combined with the deformation of the rubber matrix, counteract the loads caused by the internal pressure acting on the inner surfaces of the pipe. This results in minimal axial deformation of the pipe under internal pressure, thereby achieving balanced performance for the pipe.

**Figure 18:**
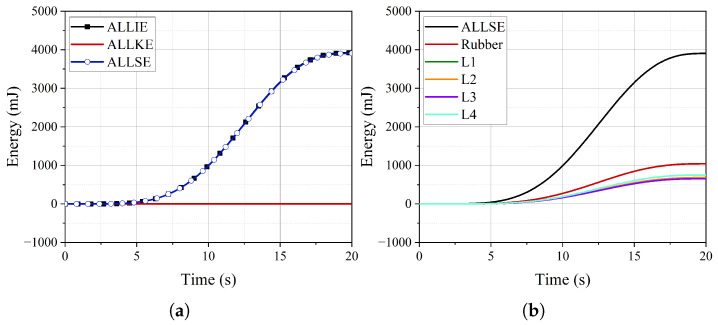
The time–history curves of energy: (**a**) the proportion of internal energy (ALLIE), strain energy (ALLSE), and kinetic energy (ALLKE) (**b**); the strain energy of rubber and fiber-reinforced layers.

The time–history curves of internal energy (ALLIE), strain energy (ALLSE), and kinetic energy (ALLKE) are illustrated in Figure 18a. Throughout the pressurization process, the internal energy curve remains notably smooth, while the kinetic energy consistently stays at a low level. For instance, at six seconds, the ratio of kinetic energy to internal energy is 0.56%, which satisfies the requirements for quasi-static calculations. At the same time, the ratio of strain energy to internal energy is 99.56%, indicating that nearly all the work imposed by the internal pressure is converted into the strain energy of the FRR pipe. Consequently, the strain energy curve directly reflects the contributions of the rubber and each fiber-reinforced layer to the strain energy of the FRR pipe.

Figure 18b and Table 4 illustrate the strain energy distribution of the components and their ratios to the total strain energy. The fiber-reinforced layers contribute 73.09% of the overall strain energy. Within these layers, the contributions of L1, L2, and L3 to the strain energy decrease with increasing radius. The contribution of the outermost layer and the innermost layer to the strain energy is equal, accounting for 19.34%. The rubber accounts for 26.91% of the total strain energy. As mentioned in reference [20], although the elastic modulus of the polymer is low compared to the fiber-reinforced layer, its significant volume ratio stands out. Therefore, the influence of the rubber matrix must be considered when establishing theoretical or numerical models to evaluate the balance performance of FRR pipes.

**Figure 19:**
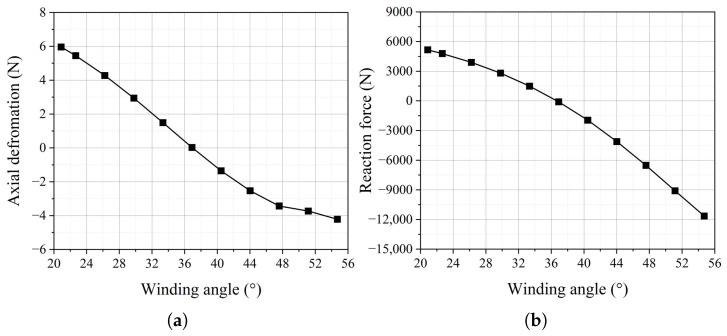
The axial deformation and reaction force of the pipe when the fiber winding angle changes: (**a**) axial deformation; (**b**) reaction force.

The dependence of axial deformation on the fiber winding angle in the FRR pipe under maximum working pressure is a critical issue worthy of investigation. According to the difference (17.8°) between the optimal winding angle (54.7°) proposed in the literature [21] and the balanced winding angle (36.9°), one-fifth of this difference is taken as the variation step for the sensitivity analysis of axial deformation. Based on this, using 36.9° as the baseline, the winding angle is varied within the range of ±17.8° to conduct the sensitivity analysis of axial deformation. Additionally, the reaction force at the end plate of the FRR pipe under maximum working pressure is calculated. As shown in Figure 19, when the fiber winding angle is 36.9°, the pipe is in a balanced state, and the reaction force is only 90.4 N. However, when the fiber winding angle deviates from the balanced winding angle, both axial deformation and reaction force increase significantly. When the winding angle varies by +3.56° and −3.56°, the axial deformation of the pipe is 44.93 and 49.86 times, respectively, compared to the balanced state. Additionally, the reaction force of the pipe is 21.65 and 16.51 times, respectively. When the winding angle is 54.7°, the axial deformation and reaction force of the pipe sharply rise to 4.21 mm and 11,648.1 N, which are 140.33 times and 128.85 times those at the balanced winding angle, respectively. These results indicate that pipes with balanced winding angles exert minimal displacement and additional force on the connected pipeline system under maximum working pressure.

### 4.2. The Axial Stiffness

**Figure 20:**
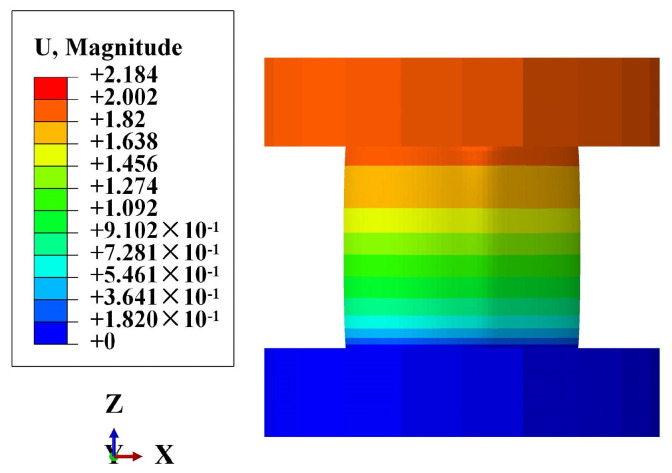
Deformation nephogram of the pipe under 1.5 MPa internal pressure.

Table 5 presents the numerical results and experimental mean values of the axial stiffness under different internal pressures. The maximum error observed is 6.16%. This discrepancy can be attributed not only to the manufacturing process but also to the experimental procedure. During testing, the accuracy of the pressure adjustment by the booster pump may cause a deviation between the actual internal pressure and the target internal pressure. Additionally, after pressurizing, the valve between the pipe and the pressurizing system is closed, leading to internal pressure fluctuations due to volume changes under axial load. In addition, the axial stiffness increases with increasing internal pressure. Notably, when the internal pressure is 1.5 MPa and axial compression is applied, the pipe exhibits an expansion phenomenon, as shown in Figure 20, which is consistent with the experimental observations in Figure 8.

**Figure 21:**
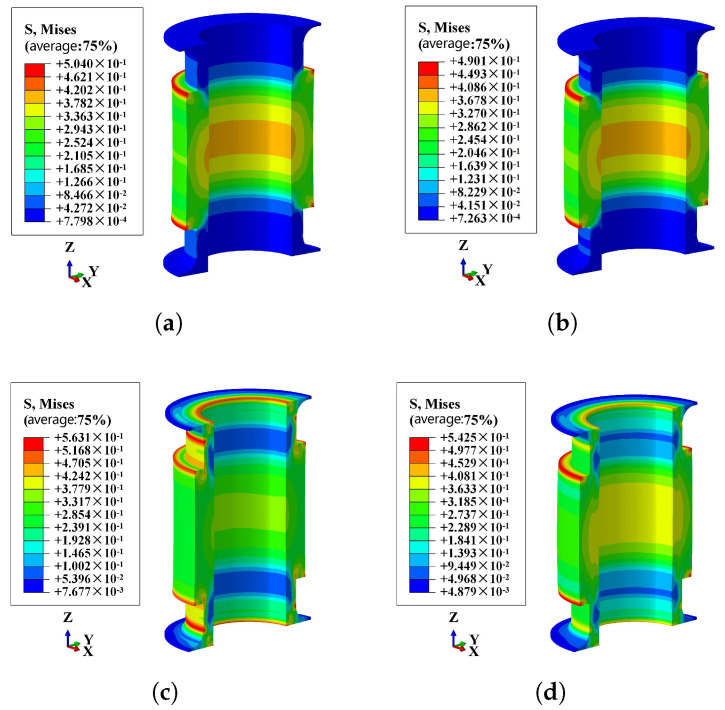
Mises stress nephogram of the pipe under different internal pressure: (**a**) the internal pressure is 0MPa and under tensile load; (**b**) the internal pressure is 0 MPa and under compressive load; (**c**) the internal pressure is 1.5 MPa and under tensile load; (**d**) the internal pressure is 1.5 MPa and under compressive load.

Based on the presence or absence of internal pressure, the Mises stress distribution in FRR pipes can be categorized into two scenarios. Figure 21a,b illustrate the stress distribution in the straight portion of the pipe when there is no internal pressure on the inner surfaces of the pipe and only an axial load is applied. In the presence of internal pressure, however, the stress distribution in the rubber near the end plates becomes more complex. This complexity arises from the interaction between the end effects and the axial load, as shown in Figure 21c,d.

**Figure 22:**
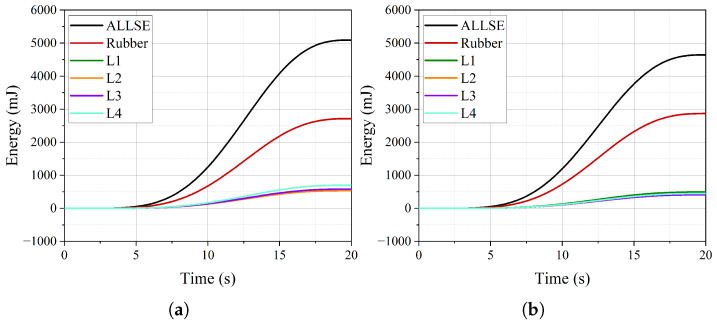
The strain energy curve of each component of the pipe under axial load when the internal pressure is 1.5 MPa: (**a**) tensile load; (**b**) compressive load.

Figure 22 and Table 6 illustrate the strain energy of the components under an internal pressure of 1.5 MPa, subjected to axial tensile and compressive loads. When the pipe is in a tensile state, the strain energy proportion of L4 is slightly higher, indicating that it bears a greater tensile load compared to the other three fiber-reinforced layers, which share similar tensile loads. In the compressed state, the strain energy distribution among the layers is relatively uniform. For the rubber, the strain energy proportions under tensile and compressive states are 53.32% and 61.76%, respectively. These proportions are at least twice as large as those calculated in Table 4, further substantiating the significant role of the rubber matrix, which cannot be neglected.

**Figure 23:**
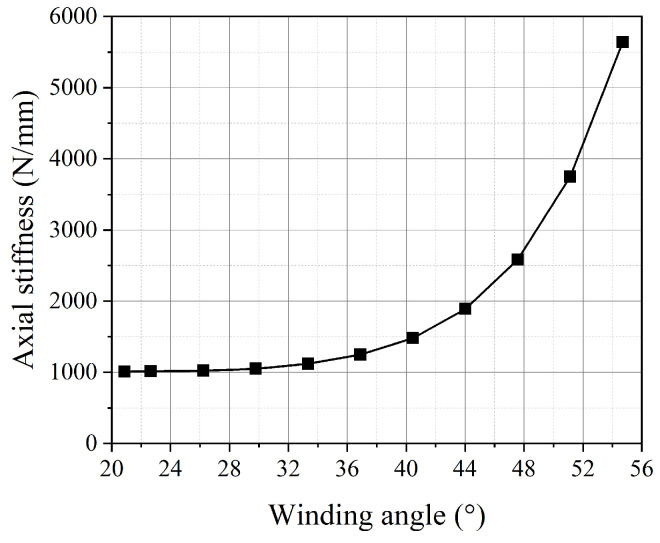
The axial stiffness of the pipe with different fiber winding angles.

The variation in the fiber winding angle affects the axial stiffness of the pipe, as shown in Figure 23. To ensure that the study focuses on a single variable, the internal pressure of the pipe is set to 1.5 MPa. When the winding angles reach 20.88° and 54.7°, the axial stiffness of the pipe decreases by 19.2% and increases by 351.71%, respectively, compared to the balanced winding angle. This indicates that the axial stiffness of the pipe is not at its minimum when the pipe is in a balanced state. The reinforcing fibers can be regarded as springs. The closer the spring direction is to the axial direction, the greater the axial stiffness it provides to the pipe. Therefore, in the design and optimization process of the pipe for practical applications, there is a trade-off between the balanced performance of the pipe and its axial stiffness.

## 5. Conclusions

In this paper, for the first time, the balanced performance of the FRR pipe and the axial stiffness of the balanced pipe are numerically simulated, considering the nonlinearity of rubber materials and the interaction between rubber and reinforcing fibers. The reliability of the numerical model is verified through experiments. Additionally, the effects of the fiber winding angle on the balanced performance and axial stiffness of the pipe are discussed. The main conclusions are as follows:

1. The balanced performance of the FRR pipe is achieved through the combined effects of the elongation and rotation of the reinforcing fibers, as well as the deformation of the rubber matrix.
2. When the pipe is in a state of balanced performance, its axial stiffness exhibits a nonlinear increase with increasing internal pressure. When the internal pressure is 1.5 MPa, the axial stiffness of the pipe increases by 33.9% compared with that when the internal pressure is 0 MPa. Furthermore, the end effects induced by internal pressure result in more stress concentration areas within the pipe compared to the condition without internal pressure.
3. The winding angle of the fiber-reinforced layer significantly affects both the balanced performance and the axial stiffness of the pipe. However, the trends in these mechanical properties differ. When the winding angle changes by +3.56° and −3.56°, the axial deformation of the pipe is 44.93 times and 49.86 times that in the balanced state, respectively. Correspondingly, the reaction force of the pipe is 21.65 times and 16.51 times that in the balanced state, respectively. However, as the winding angle decreases, the axial stiffness of the pipe tends to decrease. Therefore, for customized designs, it is essential to consider both properties comprehensively when selecting the fiber winding angle.
4. The quasi-static analysis indicates that the strain energy in the rubber matrix significantly contributes to the balanced performance and axial stiffness calculations for the pipe. During the balanced performance simulation, the strain energy of the rubber matrix accounts for 26.91% of the total strain energy. However, during the axial stiffness calculation, this value more than doubles. Consequently, the role of rubber is crucial and should not be underestimated in studies addressing the balanced performance and axial stiffness of FRR pipes.

The numerical model proposed in this paper can further guide the design of more complex FRR pipes and can also serve as a reference for engineers studying the balanced performance of other FRP composite pipes. In future research, we will focus on determining the balanced winding angle of the fibers using numerical methods prior to manufacturing, aiming to reduce costs and enhance manufacturing efficiency.

## Figures and Tables

**Table 1:** Manufacturing parameters of test samples.

Manufacturing Parameter	Value
Length (mm)	166
Inner diameter (mm)	65
External diameter (mm)	100
Innermost fiber-reinforced layer diameter (mm)	73
Outermost fiber-reinforced layer diameter (mm)	80
Number of layers of fiber-reinforced layer	4
Fiber winding angle (°)	±36.9

**Table 2:** The discrepancy between the actual measurement results and the manufacturing parameters of test samples.

Test Sample	Length (mm)	Discrepancy in Lenth (mm)	External Diameter (mm)	Discrepancy in External Diameter (mm)
1	166.1	+0.1	102.23	+2.23
2	165.8	−0.2	104.14	+4.14
3	166.2	+0.2	105.73	+5.73
4	166.3	+0.3	100.32	+0.32

**Table 3:** The axial deformation of the test samples under maximum working pressure.

Test Sample	Axial Deformation (mm)
1	−0.05
2	0.25
3	0.35
4	−0.05

**Table 4:** Strain energy of each component.

	Rubber	L1	L2	L3	L4
Strain energy (mJ)	1043.89	750.24	681.04	654.15	750.49
Ratio	26.91%	19.34%	17.55%	16.86%	19.34%

**Table 5:** The numerical results and experimental mean values of the axial stiffness under different internal pressures.

Axial Stiffness (N/mm)			
Internal Pressure (MPa)	Experimental Mean Value	Numerical Result	Percentage
0	933.02	932.02	−0.11%
0.5	1077.31	1040.98	−3.37%
1	1201.41	1146.35	−4.58%
1.5	1330.19	1248.24	−6.16%

**Table 6:** Strain energy of each component.

Load	Strain Energy and Ratio	Rubber	L1	L2	L3	L4
Tension	Strain energy (mJ)	2714.75	557.08	537.31	581.48	700.91
	Ratio	53.32%	10.94%	10.55%	11.42%	13.77%
Compression	Strain energy (mJ)	2867.62	492.86	426.74	409.42	446.81
	Ratio	61.76%	10.61%	9.19%	8.82%	9.62%

## Data Availability

The original contributions presented in the study are included in the article, further inquiries can be directed to the corresponding author.
